# A malaria transmission-directed model of mosquito life cycle and ecology

**DOI:** 10.1186/1475-2875-10-303

**Published:** 2011-10-17

**Authors:** Philip A Eckhoff

**Affiliations:** 1 Intellectual Ventures Laboratory, 1600 132nd Ave NE, Bellevue, WA 98004 USA

## Abstract

**Background:**

Malaria is a major public health issue in much of the world, and the mosquito vectors which drive transmission are key targets for interventions. Mathematical models for planning malaria eradication benefit from detailed representations of local mosquito populations, their natural dynamics and their response to campaign pressures.

**Methods:**

A new model is presented for mosquito population dynamics, effects of weather, and impacts of multiple simultaneous interventions. This model is then embedded in a large-scale individual-based simulation and results for local elimination of malaria are discussed. Mosquito population behaviours, such as anthropophily and indoor feeding, are included to study their effect upon the efficacy of vector control-based elimination campaigns.

**Results:**

Results for vector control tools, such as bed nets, indoor spraying, larval control and space spraying, both alone and in combination, are displayed for a single-location simulation with vector species and seasonality characteristic of central Tanzania, varying baseline transmission intensity and vector bionomics. The sensitivities to habitat type, anthropophily, indoor feeding, and baseline transmission intensity are explored.

**Conclusions:**

The ability to model a spectrum of local vector species with different ecologies and behaviours allows local customization of packages of interventions and exploration of the effect of proposed new tools.

## Background

Malaria is transmitted by the blood feeding of infectious female *Anopheles *mosquitoes, and understanding mosquito ecology and population dynamics can inform how best to defeat malaria. Malaria is an important global health issue, causing over half a billion cases and on the order of one million deaths a year [1], and is the focus of a global eradication campaign announced in 2007. Basic vector ecology is a fundamental driver of transmission patterns, and changes in land usage [2] or land modification can dramatically change transmission for better or worse. The growing urbanization in Africa is a powerful current example of such phenomena [3]. Climate and weather affect larval development and parasite maturation within the infected mosquito, and spatial models are able to predict malaria prevalence based primarily on climate details in the absence of interventions [4]. This climate-driven predictability has broken down more recently, possibly due to more widespread interventions such as insecticide-treated bed nets [5], but predictive modelling for global eradication incorporates these geographic effects on baseline transmission. Geographic variation and spatial effects become increasingly important as heterogeneity in transmission allows malaria to persist in some areas while other areas achieve elimination but remain at risk of reintroduction [6].

A successful global eradication campaign will include substantial vector control components, and mathematical models for planning eradication will benefit from accurate and robust representation of the basic vector transmission ecology in each area of interest as well as the ability to incorporate interventions both singly and in combination. Vector population dynamics exhibit latencies such as the time required for sporogony. Spatial processes include vector oviposition, larval habitat, host-seeking, and migration. For aptly modelling eradication, representation of the steady state is not sufficient; elimination-predictive models may need to be accurate at very low prevalence. Finally, models must address sensitivity of results to model parameters and assumptions whenever presenting possible routes to eradication.

Mathematical modelling of the vector-borne transmission of malaria dates back to the early dynamical models of Ross and Macdonald [7,8], the classical assumptions of which have been clearly exposited [9]. Next steps in vector modelling included cyclical feeding models, which were easier to parameterize from field data and more accurately tracked the mosquito life cycle [10,11]. Other models emphasize the effect of rainfall and temperature correlations to transmission [12], or compute the Entomological Inoculation Rate (EIR) driven by human infectiousness [13]. Recent work has focused on the effect of hydrology on larval habitat and vector prevalence [14], and on vector population dynamics [15]. Other groups have built comprehensive simulations for both the vector transmission dynamics and within-human parasite dynamics [16-19]. Vector population models have been constructed for other vector-borne diseases, such as dengue [20].

The present work introduces a vector model which has detailed vector population resolution for near elimination phenomena, tracks explicit latencies of larval development and sporogony, implements closed-loop population dynamics, and can implement a wide variety of vector control interventions in combination. Careful attention is given to vector behaviours, such as host preference and feeding locations, and the effects of these parameters on intervention effectiveness are explored. Alternative implementations of this model are discussed, along with parameter sensitivities. The present model is then exercised on several issues of local elimination for simulations based on transmission patterns for a single-location in Tanzania, varying baseline transmission and vector bionomics, and key results are discussed.

## Methods: model design

### Aquatic habitat

Available larval habitat is a primary driver of local mosquito populations, and different mosquito species can have different habitat preferences, with utilization of an ecological niche driving speciation in some cases [21]. Classifications of larval habitat include temporary, permanent or semi-permanent [22], and some species, such as *Anopheles gambiae ss *and *Anopheles arabiensis *will share an ecological niche for larval habitat [23]. Humans can affect available habitat through terrain changes which affect hydrology, through agricultural practices, such as rice cultivation [2], or through creating or eliminating standing water. Remote sensing through satellite imagery is becoming a powerful tool for mapping vector ecology [2,23], and this trend will most likely continue to increase as eradication planning drives increasing data requirements. Several detailed models already exist of habitat and the impact of rainfall, temperature, humidity, and soil quality [14,24,25].

Rainfall and humidity can strongly affect available larval habitat [14,22,23], although this depends on the mosquito species and its habitat preference. In fact, preference can be more specific than the species level, as *Anopheles funestus *exhibits differences in population responses to rainfall which are correlated with chromosomal diversity [26]. Rainfall, rather than habitats with water, is best correlated with numbers of *An. gambiae s.l*. This effect is not as strong as it is for culicines, nor is it universal, since *An. gambiae s.l*. have been found in stable aquatic habitats [22]. Even *An. funestus*, which prefers more semi-permanent larval habitat, has a rainfall dependence in its larval habitat [27], partly due to vegetation on edges of water [26] and the interaction of rainfall with agricultural schedules for crops such as rice.

**Figure 1:**
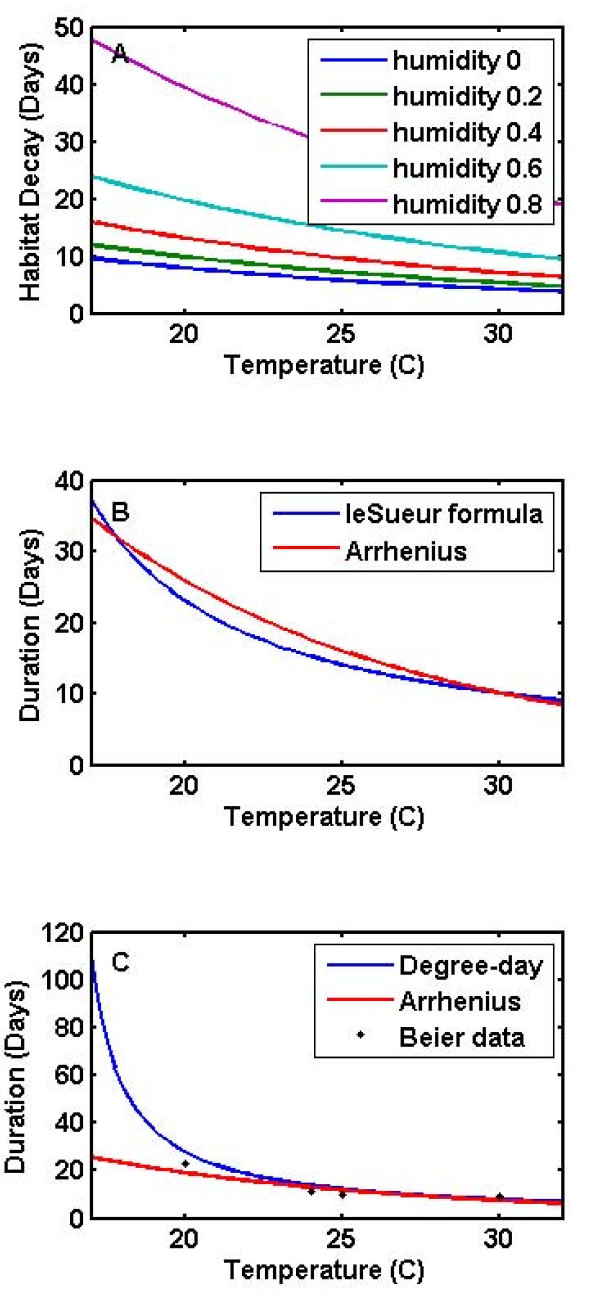
**Effects of climate and weather on vector populations**. a) Effect of temperature and humidity on time constant τ**_temp _**for temporary rainfall-driven larval habitat. The habitat decay is faster for warmer and drier weather. b) Temperature effects on duration larval development, with the functional form from [4] and the present Arrhenius formulation. c) Temperature effects on duration of sporogony. The traditional degree-day formula and the present Arrhenius function are plotted, along with Beier's data from [49].

In the present model, different models for larval habitat are developed for temporary, semi-permanent, permanent, and human-driven habitats. Temporary habitat H_temp _in a grid of diameter D_cell _increases with rainfall P_rain _and decays with a rate τ_temp _proportional to the evaporation rate driven by temperature T (K) and humidity RH:

$$H_{temp}+=P_{rain}K_{temp}D_{cell}^{2}-H_{temp}\left(\frac{\Delta t}{\tau_{temp}}\right)$$

$$\frac{1}{\tau_{temp}}=\left(5.1x10^{11}Pa\right)e^{\frac{-5628.1K}{T_{K}}}k_{tempdecay}\sqrt{\frac{.018kg∕mol}{2\pi RT_{K}}}\left(1-RH\right)$$

in which the exponential results from the Clausius-Clayperon relation, the root is from the expression for vapour evaporation rates due to molecular mass given a partial pressure, and the constant is the Clausius-Clayperon integration constant multiplied by a factor k_tempdecay _to relate mass evaporation per unit area to habitat loss. The value of k_tempdecay _is initially chosen to set the habitat half-lives near 1 day for hot and dry conditions and 2-3 weeks for more typical tropical conditions. The variation in τ_temp _with temperature T and humidity RH can be seen in Figure 1a. Semi-permanent habitat increases with a constant K_semi _D_cell_^2^P_rain _and decays with a longer time constant τ_semi_. Permanent habitat is fixed at K_perm _D_cell_^2^, and human population-driven habitat is calculated as population N* K_pop_. The values of k_tempdecay_, τ_semi_, K_semi_, and K_temp _can be fit to local data on vector abundance by species over time or to local data on EIR to tailor a simulation to a specific setting.

Larval development and mortality rates are affected by a variety of factors including weather and densities of other larvae. Climate and weather affect not only larval habitat availability but also larval development rates and larval mortality [15]. The duration of larval development is a decreasing function of temperature [4], and the present model replaces earlier mathematical formulations [4] with an Arrhenius temperature-dependent rate a_1_exp(a_2_/T_K_) as seen in Figure 1b. In some cases, this temperature-dependent rate must be modified by local larval density [28], although the presented results do not include such a modification. Rainfall and temperature then combine through habitat creation and larval development to create varying local patterns of distribution by larval instar [23], and larval mortality and development duration determine pupal rates [29].

Heavy rainfall can directly kill larvae by dislodging them from habitat and causing them to dry out [28]. Other factors increasing mortality are cannibalism of 1^st ^instar larvae by 4^th ^instar larvae and overpopulation of larval habitat acting to reduce food availability.

**Figure 2:**
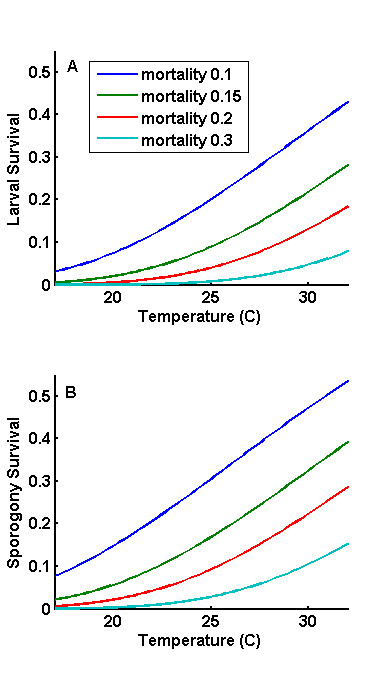
**Intermediate outputs which affect vector population or disease transmission dynamics**. a) Larval survival of development as a function of temperature and larval mortality. Survival is from successful egg hatch to emergence; survival from oviposition would be significantly lower. b) Adult survival of sporogony as a function of temperature and adult mortality. At lower temperatures, mosquitoes spend longer in each progress queue and the overall effect of a daily mortality is greater. Adult mortality can be artificially increased through interventions such as bed nets, insecticide spraying, and baited traps.

The present model includes this preferential survival of older larvae during overpopulation conditions by only treating as viable those new larvae, which do not cause the larval population to exceed capacity. If capacity shrinks so that the existing population exceeds available capacity, mortality is increased by the degree of overpopulation. With all these factors taken together, about 2-8 percent of larvae typically survive egg to adult [28]. The present model includes a daily larval mortality, which translates into a probability of survival of larval development as a function of temperature and mortality rate presented in Figure 2a. The larval survival plotted is from egg hatch to emergence, not from oviposition to adult maturity, which is significantly less due to egg survival and death during the immature phase.

### Improved cohort model

**Figure 3:**
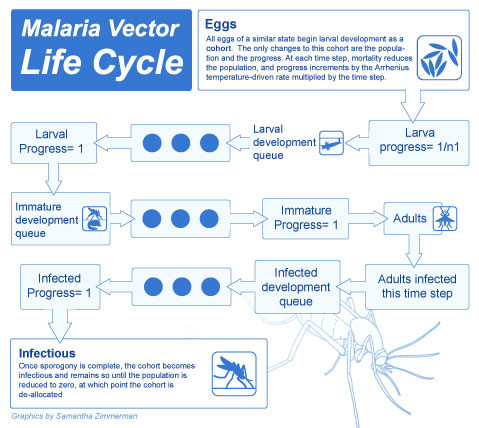
**Vector development state space**. All eggs of a similar state (species, gender, habitat, ***Wolbachia ***type) hatching in a time step begin larval development as a cohort. The only changes to this cohort are the population and the progress, and each time step, mortality reduces the population and progress increments by the Arrhenius temperature-driven rate multiplied by the time step. The progress added can vary depending on the daily temperature and is not constrained to be constant or an integer number of total days, so n1 would be the total development period at the mean temperature of the first time step. When progress through development is complete for a cohort, emergence occurs, and the cohort begins the latency to blood feeding as immature emerged adults. This latency can last for several hours up to several days, at which point the cohort begins the cycle of blood feeding. Adults infected in a time step are removed from their cohort and a new cohort is created for newly infected adults. This new cohort then proceeds through the infected development queue, with mortality reducing the population and temperature-dependent incrementing of progress. Once sporogony is complete, the cohort becomes infectious and remains so until the population is reduced to zero, at which point the cohort is de-allocated.

There are different possible implementations of the basic model, each with different computational efficiencies, resolutions, and flexibilities. Possible implementations include a modified cohort simulation, a cohort simulation with explicit mosquito ages, a simulation of every individual mosquito in the population, as well as a simulation of a sampled subset of mosquitoes to represent the population as the whole. The basic model is presented in the context of the modified cohort simulation with explanations of the modifications for individual mosquitoes. In the modified cohort simulation, rather than representing the entire population by three compartments for susceptible, latently infected, and infectious mosquitoes, the simulation dynamically allocates a cohort for every distinct state, and the cohort maintains the count of all mosquitoes in that state. This allows temperature-dependent progression through sporogony as described below, even with a different mean temperature each day, with no mosquitoes passing from susceptible to infectious before the full discrete latency. For the cohort simulation with explicit ages to allow modelling of senescence, mosquito age is part of the state definition, and many more cohorts are required to represent the population. The overall progression of cohorts or individual mosquitoes through different states is outlined in Figure 3.

### Immature mosquito populations

Upon emergence, there is a period of hours to days before bloodmeal-seeking begins [30]. This period is represented in the model as a fixed latency, during which predators and interventions such as outdoor spraying can still cause mortality. At the end of this immature interval, before the start of host-seeking, female mosquitoes mate. Male mosquitoes are included in the simulation to allow simulation of the mosquito population genetic structure, as well as interventions and phenomena such as release of modified males or mosquitoes with *Wolbachia *infection. Each female is mated once, with fertility only if the male is not sterile and there is no cytoplasmic incompatibility due to *Wolbachia *type [31]. Mating outcomes are based on the current distribution of male mosquitoes. Sterile-mated females will blood-feed, but do not produce viable eggs.

### Adult mosquito populations

**Figure 4:**
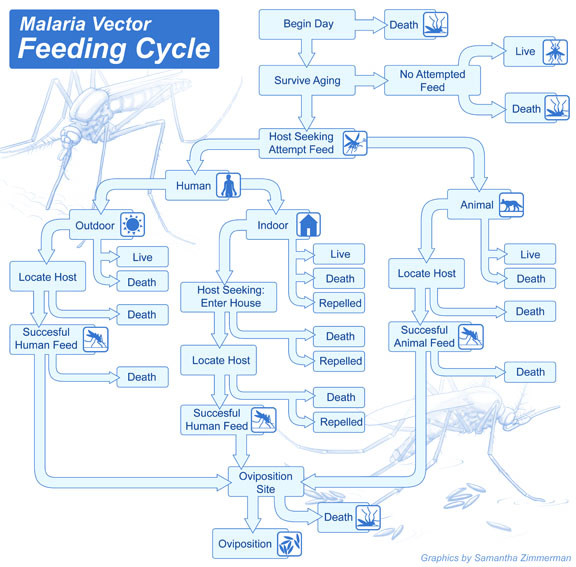
**Calculation of outcomes for each mosquito every time step in the presence of combined interventions**. Each choice has a defined probability, and the conditional probabilities can be summed for each overall possible outcome as described in the Appendix. Bed nets can kill or not, and vector feeding time can be adjusted to change the proportion of bites during the period protected by nets. Indoor feeding and resting can be split by adding in an additional decision fork after indoor and outdoor feeds. After a successful indoor feed, a mosquito must make it to an oviposition site alive to lay eggs and survive. Closed loop egg-laying allows interference by interventions to eventually limit population sizes. Individual resolution of the human population ensures that only those infectious mosquitoes that successfully pass through a gauntlet to get to a human successfully can transmit infection.

Host-seeking and blood-feeding are key aspects of the reproductive life of an adult female *Anopheles*, and these are also the key aspects for malaria transmission. After completion of post-emergence maturation, female adults enter a cycle of feeding and egg-laying which will consume the rest of their lives. Female Anopheles mosquitoes bloodfeed every 1/f = 2-4 days [11], and in the model, a fixed proportion (= fΔt) of all mosquitoes in a state cohort attempt to feed during a time step Δt. Subsequent versions of this model include a state with a timer for blood feed processing, which replaces the draw for fraction feeding each time step. The total number of mosquitoes in a cohort that attempt to feed during a time step are then stochastically sorted into a variety of outcomes depending on vector behaviour, host availability, and interventions, such as insecticide-treated nets (ITN) and indoor residual spraying (IRS) as described in Figure 4.

Possible outcomes of an attempted feeding include death, survival without feeding, successful feed on a human, or successful feed on an animal. A binary decision tree is created for progress through a feeding cycle as seen in Figure 4. If a feed is attempted, the first branching point is the choice of host type depending on the vector host preference, and if human is selected, the location of feeding is chosen based on the vector indoor feeding probability. Each possible choice is thus conditional on arriving at that stage of the decision tree, allowing simpler definitions of efficacy. Blocking efficacy of nets is specified as the probability that a net blocks a feed, given an attempted indoor feed on a protected human, rather than the reduction in overall successful biting. This binary structure allows logical combination of the effects of ITNs and IRS and makes it simple to add new interventions to the model. An indoor feed only occurs if the net does not block the feed and the treated net does not kill the mosquito. Mosquitoes that complete a feed are eligible to rest on an IRS-treated wall with a specified killing efficacy. Thus the effects of ITNs and IRS in the same house are not independent, and blocked feeds reduce the number of mosquitoes that arrive at the IRS section of the decision tree. Successful feeds on humans have an additional draw for whether the mosquito is infected with *Plasmodium *or not which depends on human infectiousness, and the conditional probability of surviving a feed on an infectious individual. En route to assembling the distribution of feeding outcomes, human biting rate and entomological inoculation rate (EIR) are calculated, including all human feeds whether or not the mosquito survives, as mosquito death can occur before, during, or after feeding, but transmission can occur even for feeds which the mosquito does not survive. The presented version of the model, with a draw for number feeding each day and without the timer for blood feed processing, calculates the outcomes for the full feeding cycle including blood meal processing and oviposition survival, but assembles these into outcomes in a single calculation with eggs laid that time step for those feeds. The detailed equations for feeding outcomes are contained in Additional File 1.

The effect of a local mosquito species population on disease transmission depends on several species-specific characteristics. Among the most important is anthropophily (p_humanfeed_), the fraction of bites, which are taken on a human host, or human blood index [32,33]. Indoor versus outdoor feeding and resting, represented as an probability of feeding indoors (p_indoorfeeding_), is another important behaviour of the local mosquito population, especially when indoor interventions such as IRS and ITN's are introduced. Vectors which feed predominantly indoors can be decimated by these interventions, while those species which feed outdoors will not experience the same applied mortality. Indoor feeding and resting are not necessarily equal for a mosquito species, and this would be simple to implement in the present outcome calculation structures. In the presented sample simulations, indoor feeds are associated with indoor resting and outdoor feeds with outdoor resting, although a given species may have a mixed proportion of indoor and outdoor feeds.

The most important factor for baseline transmission is the adult mortality [9,10], which can be calculated per day or per feeding cycle. Mosquitoes also experience additional mortality at high temperatures with low humidity [4]. The formula of Martens for daily survival to a temperature-dependent mortality rate, with T in Celsius, is approximated as (.001e^(T-32)^), which does not have the mathematical pathologies at the roots of Martens' polynomial. Mosquitoes do exhibit age effects and senescence in laboratory settings, and senescence has been observed in field studies as well [34]. In fact, mosquitoes have not been found in the field having taken more than 14 feeds [11]. These possible effects are studied by adding age to the state space, which results in a much larger number of cohorts, and adding an age-dependent mortality rate to the standard daily or feeding cycle mortality.

In the cohort model, the number of eggs laid per time step is calculated from the number of successful feeds on humans and animals occurring in that time step, with corrections for number of eggs per feeding type. There is no delay currently in the present model for egg-production, and the population growth dynamics are constrained by the days between feeds and full larval-development latencies. However, oviposition timers can be incorporated both in the individual-mosquito based simulations and in the cohort model. Determining the number of eggs from successful blood feeds allows second order effects of interventions on the mosquito population to be captured, which is not possible in models which utilize a pre-determined temporal pattern for emergence rate of mosquitoes.

### Infection

A bite on an infected human can result in mosquito infection with *Plasmodium*, with a probability of infection dependent on a variety of human and mosquito factors [35-44]. In general, human infectivity tends to increase with gametocyte densities in a typical blood meal of 1-3 μl [35], but this can be reduced by high parasitaemia provoking an inflammatory cytokine response [45,46], by age and immunity of the human host [13], and by gametocyte-killing drug treatments [47]. Once within the mosquito gut, *Plasmodium *progresses through several stages of development in the mosquito finally resulting in sporozoites within the salivary glands which can infect human hosts [48]. The mosquito attempts to avoid infection through various defenses against *Plasmodium *gametocytes [49] and melanotic encapsulation of its oocysts [50].

The effect of weather and climate on malaria transmission is seen again in temperature-dependent latencies in sporozoite development [4,49,51,52]. The development times are seen in Figure 1c, and the corresponding survival probabilities are plotted in Figure 2b. These are included in traditional continuous compartmental models as a factor for mosquito survival of this latency, which is multiplied by the rate of change in infectious mosquitoes. This effect can be implemented in cyclical model as a changing probability of surviving incubation with fixed probability of surviving a feeding cycle [53]. This cohort implementation avoids instantaneous transport from susceptible to infected status, even scaled appropriately for steady state. Steady states are rare to non-existent in malaria transmission: seasonality in temperature and rainfall changes vector population sizes and infection rates, monthly rainfall for the same month varies from year to year, and human population infectiousness may not be at the same level at the same time each year, all of which may affect the impact of interventions as a function of their timing. Therefore, it is important that full latencies are enforced, with infectious mosquitoes not appearing until completion of the intervening stages.

Progress towards infectiousness is included as a state variable, and the infection state variable is not changed from infected to infectious until the progress state variable reaches completion. Enforcement of larval and immature latencies similarly captures the dynamics for population growth. Mosquitoes of the same state infected the same evening become a new state cohort in the simulation, and each time step, progress towards infectiousness is incremented by the temperature-dependent rate. At infection, the number infected is subtracted from the population of the uninfected cohort, and a new cohort is allocated with the newly infected population and zero progress towards sporogony. Upon completion, either the cohort is either merged with an identical-state infectious mosquito state cohort, or maintained separately if no identical state likely exists, as in the case of age-tracking.

Outcome probabilities can change in response to infection status. Sporozoite infection of the salivary glands can result in increased feeding mortality when infectious [54,55]. Fecundity can be affected as smaller egg-batch size is observed due to maturing oocysts [56] but not salivary-gland sporozoites [57]. Once infectious, a mosquito's bites have a probability of infecting a human host, whether the mosquito survives the feed or dies during or after the feed. Probability of human infection from a sporozoite-positive bite can be calculated from field data or laboratory experiments, with a value of approximately 0.5 per bite probably being reasonable [58].

### Interventions

Simulations of vector populations in the absence of interventions, such as bed nets, are important, but the purpose of the present model is to evaluate the effects of interventions singly and in combination, especially in the global eradication context. Key issues include incorporation of the effects of each of the full spectrum of interventions to examine possible effects and determining how interventions combine their effects. Some interventions target adult female *Anopheles *feeding, and these include insecticide treated bed nets [59-61], indoor residual spraying and screening [62]. Figure 4 shows how each intervention affects the feeding cycle as discussed above and how total outcomes can be calculated when interventions are combined, with full equations in Additional File 1.

Other interventions affect the population through the larval stage, either directly with larvicides [63,64] and larval predators or indirectly through habitat destruction. Land usage modification, either intentional or unintentional, due to urbanization, agriculture, or draining swamps can have powerful effects [62,63]. Larval control options available in the model include temporary increases in larval mortality through larvicides in a subset of local habitat, repeated treatments with sufficient mortality to render a fraction of local habitat unavailable for longer intervals, or land usage reducing larval habitat long term. Depending on the option, the model either implements a temporary increase in larval mortality in a subset of the habitat carrying capacity, or proportionately reduces the habitat carrying capacity for the duration of effect.

### Individual mosquito model

In addition to state cohorts, this basic model can be implemented through simulation of every individual mosquito or simulation of a subset of individual mosquitoes to represent the full population. Each mosquito's state contains the same features as the state cohort model, with status, timers for transition to adult from immature and infected to infectious, mating status and *Wolbachia *infection, and age. An oviposition timer to enforce a fixed feeding cycle may be included as well. If mosquitoes are sampled and a subset used to represent the local population, each sample mosquito will have an associated sampling weight as well.

### Setting up simulations

Vector dynamics are simulated for single human populations well-mixed with multiple vector populations. All simulations are based upon a single-location with temperature [19] and rainfall [65] based upon lat-long (-8.5, 36.5) in Tanzania. Three local vector populations are simulated: *An. gambiae s.s*., *An. arabiensis*, and *An. funestus. Anopheles gambiae *and *An. arabiensis *are modelled to track the rainfall with the short temperature and humidity-dependent time constant, while *An. funestus *larval habitat integrates rainfall with a smaller forcing term and decays with a much slower time constant, here set to 100 days to correspond to the length of an agricultural season. The habitat scaling parameters and habitat-specific time constants were obtained by simulation of one species at a time, comparing to measurements of local EIR by species. Parameters which exhibit high uncertainty or geographic variability, such as the host preference of *An. arabiensis*, are studied over broad numerical ranges for their impact on results. A simplified human disease model is used in all simulations, with a constant latent period of 22 days from bite to infectiousness to mosquitoes, and exponentially-distributed period of infectiousness with mean 180 days. Infectiousness is a constant 0.2, without development of immunity to allow resolution of vector-specific effects. Superinfection is allowed, with a maximum of five simultaneous infections. General model and simulation-specific parameters and their values are summarized in Table 1.

**Table 1:** Model and simulation parameters

Parameter	Value used in simulations	Source, notes
Habitat scalars K_temp_	1.25 × 10^9 ^for *gambiae ss *and 11.25 × 10^9 ^for *arabiensis*	Fit to site-specific data through simulation

Habitat scalar K_semi_	6 × 10^8 ^for *funestus*	Fit to site-specific data through simulation

Habitat time constants k_tempdecay _and τ_semi_	0.05 (k_tempdecay_) 0.01/day (τ_semi_)	Fit through simulation

Larval development Arrhenius parameters a_1_, a_2_	8.42 × 10^10^, 8.3 × 10^3^	Fit to traditional curve in [4]

Incubation period Arrhenius parameters a_1_, a_2_	1.17 × 10^11^, 8.4 × 10^3^	Fit to traditional curve in [4]

Duration of immature	4 days	Not a very sensitive parameter, given the habitat fit to adult population

Days between feeds	3 days	2-4 days [11]

Human blood index	0.95 for *gambiae ss *and *funestus*, variable for *arabiensis*	[69]. The uncertain value for *arabiensis *is the focus of detailed analyses.

Indoor feeding	To explore effects, gambiae and funestus were set as highly endophilic and arabiensis was varied	

Female eggs per female oviposition	100	A more accurate value would be closer to 80

Modification of egg batch size for infection	0.8	

Adult life expectancy	10 days	[9-11]

Transmission modifier b	0.5	[58]

Mosquito infection modifier c	0.2	Will in reality depend on human infectiousness [36], here set to be uniform for simplicity

Human feeding mortality	0.1	Uncertain

Human feeding mortality for sporozoite positive	0.15	Uncertain

## Results and Discussion

**Figure 5:**
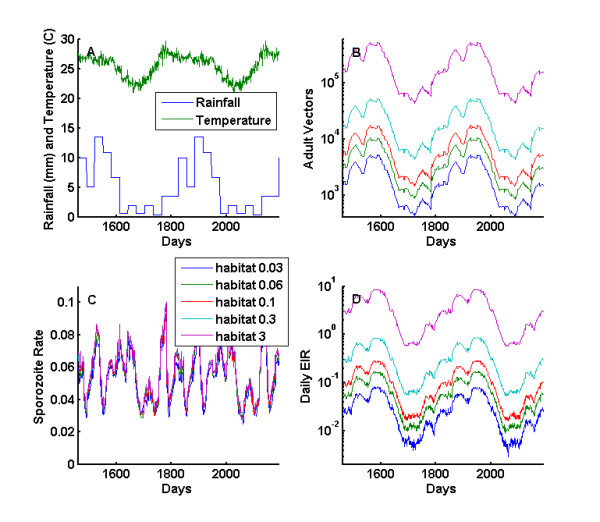
**Baseline population dynamics summed over local populations of *An. gambiae ss, An. funestus*, and *An. arabiensis *for different larval habitat multipliers**. a) Local weather will drive both the temporary and semi-permanent larval habitats. b) The adult vector population changes as a function of the scaling of the larval habitat carrying capacity, which is driven by local weather. c) The sporozoite rate of mosquito population changes in response to the changing age structure of the vector population over the course of two years. d) Daily EIR combines the adult vector population and the sporozoite rate.

Baseline vector population and transmission dynamics are simulated for the single location simulation described in Methods. The habitat scaling parameters are varied to show different baseline EIRs with the same weather-driven seasonality. The total vector population when summed across all three species is seen in Figure 5a, with the rainfall patterns and temperature in 5 b. Figure 5a shows the effect of scaling the time series of available larval habitat, Figure 5c presents the resulting sporozoite rates, and Figure 5d the entomological inoculation rate, which represents the infectious bites received per person per night. If mortality increases as a function of age, the total population numbers do not change greatly, but the sporozoite rate drops due to the suppression of the older part of the mosquito age-distribution. Note that increasing larval habitat has a second order effect on EIR by allowing the human prevalence to rise earlier in the season, which increases the infectivity of the human population to vectors.

**Figure 6:**
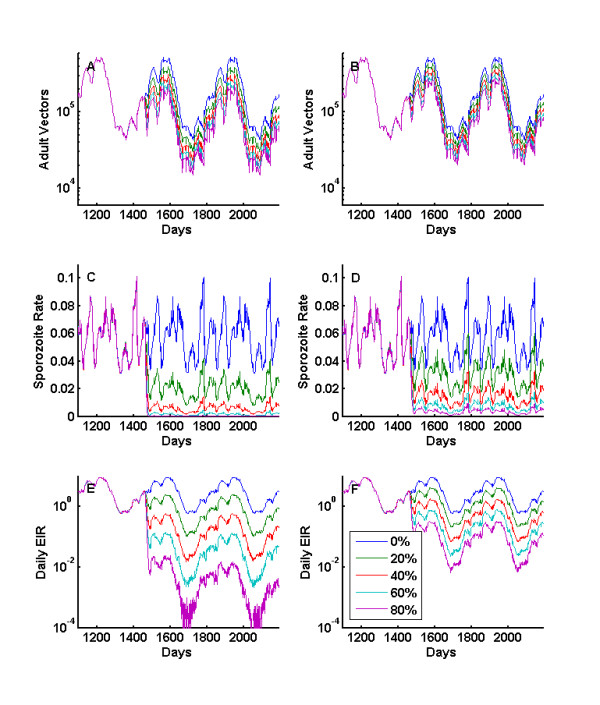
**Effects of increasing coverage with perfect IRS**. Effects of increasing coverage with perfect IRS (p**_kill, IRSpostfeed _**= 1) on a) Adult population c) Sporozoite rate e) EIR. Effects on sporozoite rate and EIR are much more dramatic than on the adult population because of the restructuring of the age distribution of the mosquito population. For most coverage levels, larval habitat remains the limiting factor in the rate of emerging mosquitoes, and number of young, unfed mosquitoes remains similar as IRS coverage increases up to a point. However, the increased feeding mortality results in a decreased life expectancy for mosquitoes, a moderate reduction in total population, but a strong reduction in mosquitoes older than 10 days. b, d, f) Repeated for IRS with (p**_kill, IRSpostfeed _**= 0.6). The effects on sporozoite rate and EIR are not as dramatic due to improved mosquito survival. The larval habitat multiplier is set to 3.0 for these simulations.

Further simulations demonstrate effects of combined vector control interventions such as insecticide-treated nets (ITN), indoor residual spraying (IRS), larval control, and space spraying. Figures 6 a, c, e show the changes in vector dynamics as coverage with perfect IRS is increased, killing all indoor feeding mosquitoes and maintained at full efficacy for the specified coverage without decay. In addition, all mosquitoes are set to feed indoors, making this an unrealistic scenario, but a useful boundary case showing the maximum possible effect. Larval habitat is set to 3.0 for all three species, and the simple human disease model is used. The detailed model outputs can be used to determine the change in entomologic inoculation rate as described in [9]. These results can be compared to field results for bed net campaigns in the presence of multiple vector species [66]. The size of the local vector population is reduced, but the effects on sporozoite rate and EIR are much more dramatic for several reasons, especially the restructuring of the mosquito population age distribution. The higher mortality results in fewer old mosquitoes in the population, which is the segment of the population with sporozoites. The older cohorts are thus responsible for the major portion of EIR but only minor portions of adult populations, human biting, and fecundity. In many cases, larval habitat remains the limiting factor in determining the number of emerging mosquitoes and interventions primarily act through adult mortality, but at high IRS or ITN coverage levels, it is possible in simulations to reduce emergence rate by limiting successful feeds. This phenomenon has been seen in high-coverage field studies [67], and the disappearance of *An. funestus *from parts of its earlier habitat as intervention coverage increases is the extreme limit of this phenomenon. The simulations are repeated for IRS with 0.6 killing of post-feeding mosquitoes and results are shown in Figures 6 b, d, f. The effects on adult vectors and sporozoite rate are reduced, and these reductions are compounded in the effect on EIR. Feeds in houses with IRS now have a 40 percent survival probability in contrast to the 0 percent survival in the previous simulations. Thus for 60 percent coverage, the survival for indoor feeds on the population is now 64 percent instead of 40 percent, and the probability of surviving three feeds rises to 26 percent from 6 percent.

**Figure 7:**
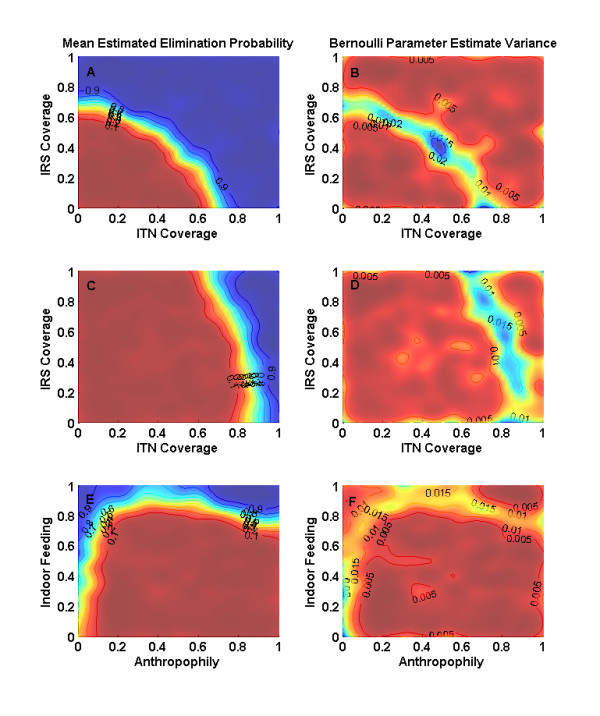
**Effects of combining IRS and ITN**. a, b) Probability of Eradication and Estimate Variance for perfect bed nets and IRS, which do not decay, for fully indoor feeding and resting mosquitoes c, d) Bed nets still prevent all nighttime feeds, but only kill 60% of mosquitoes attempting to feed. IRS kills 60% of post-indoor feeding mosquitoes in treated houses. Eradication is no longer possible in many previously possible parameter regions. e, f) Effect of varying indoor feeding and anthropophily of the ***An. arabiensis ***population for 90 percent IRS coverage with no decay of insecticide and p**_kill, IRSpostfeed _**= 1. The multiplier for larval habitat set to 1.0 for all three sets of simulations, and increasing larval habitat increases the level of coverage required, but not as dramatically as changing the adult mortality. Dark blue regions in a, c, e correspond to parameter regimes in which the estimated probability of local elimination is over 0.9 and dark red less than 0.1. Level sets for mean estimated probability of elimination and for probability estimate variance are labeled.

The effects of co-varying IRS and ITN coverage are studied and many simulations are run over sections of campaign and parameter space, with each simulation a trial for local elimination success or failure. These trials can be used to estimate the probability of local elimination as a function over campaign or parameter space. In Figure 7, coverage with IRS and ITN is varied, and the trials are assembled into plots, which map the regions of high probability of success and low probability of success. For purposes of this demonstration, both IRS and ITN kill every single relevant mosquito (p_kill, ITN _= 1 and p_kill, IRSpostfeed _= 1) and do not decay. All three mosquito species are simulated to feed indoors and take 95 percent of their blood meals on human hosts. The larval habitat scaling is set to 1.0, and the simple human disease model is used. Given these assumptions, it is not surprising that the region of success is large. Decreasing p_kill, ITN _and p_kill, IRSpostfeed _to 0.6 and maintaining bed nets at 100% blocking of indoor feeding produces the 2-D plot in Figure 7c. High coverage of bed nets still successfully locally eliminates the disease, since all feeds in this simulation occur indoors at night, but this is a quadratic effect of blocking part of the population from acquisition and transmission, while the exponential effect of increasing local mosquito mortality is drastically reduced with dramatic effects. Even with full IRS coverage and all mosquitoes feeding indoors, a reduced killing efficacy may not permit local elimination without supplemental interventions. The reduced efficacy is intended to represent an effect of insecticide resistance, and the effect on elimination has important implications for campaigns.

Changes in vector behaviours such as indoor feeding and anthropophily change the results drastically with several key implications for eradication. The previous system is rerun with IRS at 90 percent coverage with full mosquito post-feeding mortality for indoor feeds in treated-houses, and the human feeding and indoor feeding preferences of the local *arabiensis *population are varied. Changes in indoor feeding have a dramatic effect on campaign success as seen in Figure 7 e, f, as would be expected for interventions which only affect indoor feeding mosquitoes. Decreasing anthropophily is often viewed as decreasing transmission, but in the presence of high intervention coverage, decreasing anthropophily reduces the mortality during sporogony, allowing a higher proportion of infected mosquitoes to complete sporogony and thereby reducing the probability of campaign success. Feeds on animals during sporogony are safe compared to the protected human feeds and increase the probability of surviving the multiple feeds during sporogony and becoming infectious. At lower anthropophily, fewer mosquitoes become infected and infectious mosquitoes bite humans less frequently, and this effect eventually wins and probability of success increases with decreasing anthropophily below a certain value. Detailed model representations of vector behaviour help explain the failure of elimination campaigns which only targeted indoor feeding. For eradication to succeed, the full transmission cycle must be sufficiently broken, which may involve targeting outdoor-feeding mosquitoes in some areas.

**Figure 8:**
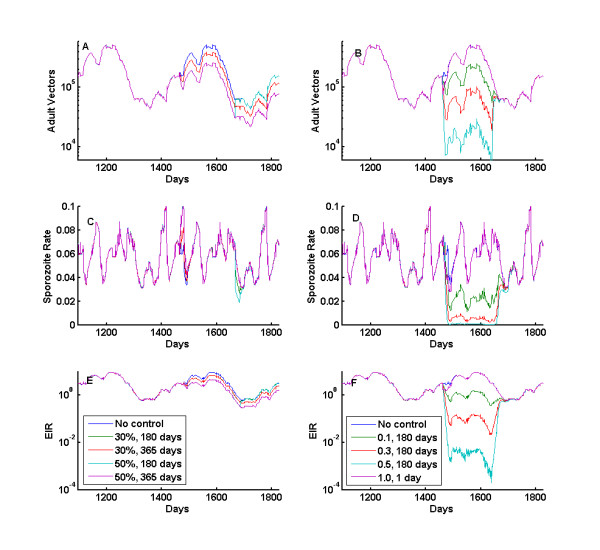
**Larval control and space spraying**. Larval control and space spraying introduced to the baseline simulations from Figure 5, with larval habitat multipliers set to 3.0. a, c, e) Larval control on the left reduces adult vector populations and EIR, but does not affect sporozoite rate at these high EIRs as the age structure of the vector population is unaffected. b, d, f) Adult mortality affects all three measures, as it reduces the number of adult vectors, and dramatically changes the age structure so that fewer mosquitoes are old enough to have sporozoites. This creates a compounded effect on EIR.

Other available but currently less-used options for vector control include larvicides and space spraying for targeting larval and adult populations, respectively. Figure 8 shows results for simulations of larval control on the left and adult-targeting space spraying on the right. In the presented simulations, larval control is simulated as a temporary decrease in the larval habitat carrying capacity for a specified duration, such as a 30 percent reduction for 180 days. This produces decreases in the adult population, but not in the adult age-structure-driven sporozoite rate, except for transients at the start and conclusion of larval control. Thus the effects on EIR tend to be linear, but this should not be ignored as larval control may be one of the only ways to target outdoor feeding mosquitoes.

In order to achieve local elimination of malaria in areas with high rates of outdoor feeding by the local vector populations, some form of control of outdoor mosquitoes may be necessary. Available options can be costly and logistically difficult, and studies of their effects can place constraints on required target efficacy, duration, and frequency of such efforts. In the model, space spraying increases the mortality for all adult mosquitoes, regardless of the stage in the feeding cycle. The artificial daily mortality probability is calculated as 1-exp(-killrate*Δt), so that a killrate of 1.0 will tend to kill approximately 63 percent of adult vectors. As seen in Figure 8, even one day of spraying with a high killrate can produce a large several week drop in EIR as it takes time for the newly emerging mosquitoes to become infected and infectious. Longer duration efforts, such as repeated spraying with a given knockdown of adult vectors each cycle, increase the daily mortality and reduce the number of adult vectors, reshape the age structure and reduce the sporozoite rate and achieve a strong multiplicative decrease on the EIR. Such maintained repetitive space spraying with non-residual insecticides may be logistically difficult, but it could provide leverage on the outdoor feeding population if other options fall through. Simulations such as these can estimate the effects on vector populations for a given input efficacy, duration, and frequency of application, providing inputs to cost-effectiveness analyses.

## Conclusions

The present model creates a flexible framework for exploring the effects of combined vector control interventions on vector population dynamics and disease transmission. Campaigns with IRS and ITN are studied and success of elimination is seen to depend on coverage and efficacy as expected, but also on mosquito behaviour. As *An. arabiensis *outdoor feeding increases, interventions which target indoor feeding become less effective. Decreasing anthropophily from unity at high coverage decreases the rate of killing mosquitoes during sporogony, initially reducing elimination success, but this returns to the expected relationship of less anthropophily increasing elimination success as anthropophily continues to decline to low levels. Climate and weather data with high spatial resolution can help predict spatial and temporal patterns of vector dynamics and assist the rational planning of regional campaigns, especially when included combined with a population transmission model [16,17,19].

Further work will exhibit the effect of vector migration and seasonality on interventions such as locally-applied larvicides. Understanding the role and scale of migration is important for estimating effect of adult vector interventions [61,67] and larval control interventions [9,59,63,64]. The model supports spatially-distributed simulations, and future work will explore the effects of human and vector migration on spatial transmission. Each individual in the simulation has a relative biting rate, which allows study of heterogeneous biting as has been done [37], and link to studies of attractiveness of humans to mosquitoes versus level of malaria infection [68]. Improvements will be made to the density-dependence effects of larval dynamics and models for habitat calculations will be improved. Other future work will model the response of vector populations to applied pressure, such as the lower anthropophily of *An. gambiae ss *in The Gambia after extended bed net pressure [32]. Changes in behavior or development of insecticide resistance require careful consideration to ensure success of a designed Eradication campaign. The effects of such changes can be seen in the above-presented results, and the present model has the flexibility to incorporate dynamic changes. The modular structure of the model and the implementation of the life and feeding cycles also make it simple to add new potential interventions to the model. Other species are easy to add provided that an applicable habitat model has been developed. Future work can couple this detailed vector transmission model to a more detailed model of human disease and immunity.

Campaigns must address the ecology and behaviour of local mosquito populations in order to ensure that sufficient resources with broad enough effects for all relevant components of the local mosquito populations are introduced. A one-size-fits-all campaign is not optimal, being wasteful in some circumstances and insufficient in others; local tailoring and design are important. Modelling can be used to estimate the risk of disease transmission given reintroduction to areas that had achieved local elimination before their neighbours [6]. Modelling at this level of detail also serves to identify basic data gaps such as local vector ecology and behaviour that must be answered to reduce uncertainty of campaign success. Numerical studies can reveal to which parameters the results of interest are most sensitive, and such parameters which are also poorly constrained by data or are highly geographically-variable can then be highlighted as important data gaps. Modelling studies can also explore the extent to which more through and extensive campaigns can overwhelm data uncertainties and achieve more robust success.

## Competing interests

The authors declare that they have no competing interests.

## Supplementary Material

Additional file 1 **Detailed equations for A malaria transmission-directed model of mosquito life cycle and ecology**. The detailed calculations of vector feeding outcomes for feeds on a single individual and on the full local human population are provided. Click here for file
