# Supplementary material for: A malaria transmission-directed model of mosquito life cycle and ecology
Source: Malar J. 2011 Oct 17;10:303. doi: 10.1186/1475-2875-10-303 (PMC3224385; doi:10.1186/1475-2875-10-303)
Supplement: Additional file 1 — Detailed equations for A malaria transmission-directed model of mosquito life cycle and ecology. The detailed calculations of vector feeding outcomes for feeds on a single individual and on the full local human population are provided. [file 1475-2875-10-303-S1.DOC]

**Detailed equations for**

**A malaria transmission-directed model of mosquito life cycle and ecology**

Philip A Eckhoff

**Individual vector feeding outcomes**

Every possible outcome in the feeding cycle is represented as a set of binary decisions with defined probabilities. Mortality rates due to natural and artificial causes are calculated and converted to probabilities of dying in a time step as follows. The exponential formulation of probability is to maintain accuracy for high kill rates for which death multiplied by a one day time step would cause numerical integration errors for other schemes.

localadultmortality=(basic mortality + aging mortality + dryheatmortality)

outdoorareakilling=(total kill rate due to artificial means over outdoor areas, e.g. outdoor spraying)

p_localadultmortality_=1-exp(-localadultmortalityΔt)

p_outdoorareakilling_=1-exp(-outdoorareakillingΔt)

Calculation of outcomes A—die without human feeding, B—survive without feeding, C—successful animal feed, D—successful feed on distracting bait, E—attempt of indoor human feed, F—attempt of outdoor human feed as described in Figure 4. Each level in this outline is a separate binary decision with a specific probability. Each path through the decision tree then has a total probability, and endpoints with the same outcome have their probabilities totaled. New interventions cause new branches in the cycle and are added to the calculations below accordingly.

- Die naturally (p_localadultmortality_)
- Survive aging (1- p_localadultmortality_)
  - Don’t try to feed (1-fΔt)
    - Killed artificially by outdoor spraying (p_outdoorareakilling_)—OUTCOME A
    - Survives without feeding (1- p_outdoorareakilling_)—OUTCOME B
  - Try to feed (fΔt)
    - Distracted by AD-OV to feed away from village (p_attraction,ADOV_)
      - Die artificially (p_outdoorareakilling_)—OUTCOME A
      - Successful feed on feeder (1-p_outdoorareakilling_)—OUTCOME D
    - Proceeds to village (1-p_attraction,ADOV_)
      - Killed by PF-V when approaching village (p_kill,PFV_)—OUTCOME A
      - Spatial repellent (1- p_kill,PFV_)p_spatialrepellent_
        - Die artificially (p_outdoorareakilling_)—OUTCOME A
        - Survive (1- p_outdoorareakilling_)—OUTCOME B
      - Passes into village (1-p_kill,PFV_)(1-p_spatialrepellent_)
        - Distracted by AD-IV (p_attraction,ADIV_)

Die artificially (p_outdoorareakilling_)—OUTCOME A

Feeds on AD (1-p_outdoorareakilling_)

Killed by PF-V returning(p_kill,PFV_ )—OUTCOME A

Successful AD feed—OUTCOME D

- - - - - Other host(1-p_attraction,ADIV_)

Feed on animal (1-p_humanfeed_)

Die artificially (p_outdoorareakilling_)—OUTCOME A

Survives

Die due to livestock-targeted pesticide (p_kill,LTP_)—OUTCOME A

Survives animal feed (1- p_kill,LTP_)

Killed by PF-V returning—OUTCOME A

Successful feed on animal—OUTCOME C

Feed on human (p_humanfeed_)

Attempted indoor feed (p_indoorfeeding_)—OUTCOME E

Outdoor feed (1- p_indoorfeeding_)

Die artificially (p_outdoorareakilling_)—OUTCOME A

Survive artificial death

No host found –OUTCOME B

Human found –OUTCOME F

Outdoor human feed state space for those mosquitoes which find a human host outdoors (OUTCOME F)

1. die during feeding (p_humanfeedingmortality_)
2. Survives human feed (1- p_humanfeedingmortality_)
   1. PF-V returning p_kill,PFV_
   2. Successful human feed (1- p_kill,PFV_)

Indoor human feed state space, for those mosquitoes that are attempting a human indoor feed (OUTCOME E), given the probability of vector trying to enter house to feed on human calculated as:

(fΔt)(1- p_localadultmortality_)(1-p_attraction,ADOV_)(1-p_kill,PFV_)(1-p_attraction,ADIV_)(p_humanfeed_)( p_indoorfeeding_)

1. Killed by PF-H (p_kill,PFH_)
2. Survives PF-H (1-p_kill,PFH_)
   1. Blocked by screens (p_block,screen_)
   2. Makes it to house (1-p_block,screen_)
      1. Dies from IRS pre-feed (p_kill,IRSprefeed_)
      2. Survives to feed(1-p_kill,IRSprefeed_)
         1. Distracted by AD-IH (p_attraction,ADIH_)
            1. Killed by IRS postfeed (p_kill,IRSpostfeed_)
            2. Survives IRS (1-p_kill,IRSpostfeed_)

Killed by PF-H leaving (p_kill,PFH_)

Escapes house (1-p_kill,PFH_)

Killed by PF-V (p_kill,PFV_)

Successful feed on feeder (1-p_kill,PFV_)

- - - 1. Looking for human (1-p_attraction,ADIH_)
         1. Nets prevent feed (p_block,net_)

Mosquito survives net (1-p_kill,ITN_)

Killed by PF-H leaving (p_kill,PFH_)

Leaves successfully (1-p_kill,PFH_)

Mosquito dies pre-feed (p_kill,ITN_)

- - - - 1. Feed occurs (1-p_block,net_)

Die during feeding (p_humanfeedingmortality_)

Completes feed (1-p_humanfeedingmortality_)

Die post-feed from IRS (p_kill,IRSpostfeed_)

Survives (1-p_kill,IRSpostfeed_)

Killed by PF-H leaving (p_kill,PFH_)

Survives PF-H (1-p_kill,PFH_)

Killed by PF-V (p_kill,PFV_)

Successful feed on human (1-p_kill,PFV_)

Outcome probabilities after effects of natural mortality p_localadultmortality_

OUTCOME A—die without human feeding

p_A_=1-p_B_-p_C_-p_D_-p_E_-p_F_

OUTCOME B—survive without feeding

p_B_=(1-fΔt)(1- p_outdoorareakilling_)+(fΔt)(1-p_attraction,ADOV_)(1-p_kill,PFV_)(p_spatialrepellent_(1- p_outdoorareakilling_)+(1-p_spatialrepellent_)(1-p_attraction,ADIV_) p_humanfeed_ (1- p_indoorfeeding_)(1- p_outdoorareakilling_)p_nooutdoorhumanfound_)

OUTCOME C—successful animal feed

p_C_=fΔt(1-p_attraction,ADOV_)(1-p_kill,PFV_)(1-p_spatialrepellent_)(1-p_attraction,ADIV_)(1- p_humanfeed_)(1- p_outdoorareakilling_)(1-p_kill,LTP_)(1-p_kill,PFV_ )

OUTCOME D—successful feed on artificial feeder

p_D_=fΔt(p_attraction,ADOV_(1- p_outdoorareakilling_)+(1-p_attraction,ADOV_)(1-p_kill,PFV_)(1-p_spatialrepellent_)p_attraction,ADIV_(1- p_outdoorareakilling_)(1-p_kill,PFV_))

OUTCOME E—attempt to human feed indoor

p_E_=fΔt(1-p_attraction,ADOV_)(1-p_kill,PFV_)(1-p_spatialrepellent_)(1-p_attraction,ADIV_) p_humanfeed_ p_indoorfeeding_

OUTCOME F—attempt to human feed outdoor

p_F_=fΔt(1-p_attraction,ADOV_)(1-p_kill,PFV_)(1-p_spatialrepellent_)(1-p_attraction,ADIV_) p_humanfeed_ (1- p_indoorfeeding_)(1- p_outdoorareakilling_)(1-p_nooutdoorhumanfound)

Indoor probabilities

Indoor outcome a—die pre-feeding

p_indooroutcome,a_ = p_kill,PFH_+(1-p_kill,PFH_)(1-p_block,screen_)(p_kill,IRSprefeed_+(1-p_kill,IRSprefeed_)(p_attraction,ADIH_(p_kill,IRSpostfeed_+(1-p_kill,IRSpostfeed_)p_kill,PFH_)+(1-p_attraction,ADIH_)p_block,net_((1-p_kill,ITN_)p_kill,PFH_+p_kill,ITN_)

Indoor outcome b—survive without feeding

p_indooroutcome,b_ = (1-p_kill,PFH_)(p_block,screen_+(1-p_block,screen_)(1-p_kill,IRSprefeed_)(1-p_attraction,ADIH_)p_block,net_(1-p_kill,ITN_)(1-p_kill,PFH_))

Indoor outcome c—die during feeding

p_indooroutcome,c_ = (1-p_kill,PFH_)(1-p_block,screen_)(1-p_kill,IRSprefeed_) (1-p_attraction,ADIH_)(1-p_block,net_)p_humanfeedingmortality_

Indoor outcome d—die post-feed

p_indooroutcome,d_ = (1-p_kill,PFH_)(1-p_block,screen_)(1-p_kill,IRSprefeed_) (1-p_attraction,ADIH_) (1-p_block,net_)(1- p_humanfeedingmortality_)(p_kill,IRSpostfeed_+(1-p_kill,IRSpostfeed_)p_kill,PFH_)

Indoor outcome e—successful human feed

p_indooroutcome,e_ = (1-p_kill,PFH_)(1-p_block,screen_)(1-p_kill,IRSprefeed_) (1-p_attraction,ADIH_) (1-p_block,net_) (1- p_humanfeedingmortality_)(1-p_kill,IRSpostfeed_)(1-p_kill,PFH_)

Indoor outcome f—successful AD feed

p_indooroutcome,f_ = (1-p_kill,PFH_)(1-p_block,screen_)(1-p_kill,IRSprefeed_)p_attraction,ADIH_(1-p_kill,IRSpostfeed_)(1-p_kill,PFH_)

**Calculation of net vector outcome probabilities**

These human feeding outcomes are clearly specific to an individual human, their housing situation and ownership and use of interventions such as bednets or screening. The terminal feeding outcomes for an attempted feed on any individual are disjoint, and mosquitoes feeding on that individual are each sorted into a single one of these outcomes, so a single random number could be drawn to sort a feeding mosquito into an outcome using the discrete cumulative probability distribution. In addition, the choice of human host is dependent on that individual’s heterogeneous attractiveness and accessibility to mosquitoes which creates a relative biting rate compared to the population average. Not all individuals in the population are necessarily added to the simulation; individuals in the population can be probabilistically sampled for inclusion in the simulation under certain conditions, and each sampled individual receives a sampling weight W_i_ equal to the inverse of the sampling rate. Sampling people allows the same model equations to efficiency simulate dynamics in both continuum and sparse regimes, with full sampling (e.g. W_i_ =1 for all) often required for sparse regimes. The individual’s relative biting rate is multiplied by this sampling weight to determine the effective “cross-section” of that individual’s feeding outcomes to the mosquito population. The outcomes for each individual can be averaged, weighted by the relative biting rate and sampling weight, to determine the discrete cumulative probability distribution for mosquitoes feeding on the population. If an individual in the simulation has a sampling weight of W_i_ and a relative biting rate of α_i_, the probability P_f,i_ that simulated individual i will be chosen by a human-seeking mosquito is then calculated as

$$P_{f,i}=\frac{\alpha_{i}W_{i}}{\sum_{i} \alpha_{i}W_{i}}$$

A mosquito attempting to feed on a human (OUTCOMES E,F) has several possible outcomes which are grouped into four categories each for indoor and outdoor feeding: survives without feeding, dies (before, during or after feed doesn’t matter to the mosquito), successfully feeds on a human, or successfully feeds on a distracting feeder, the probabilities of which sum to 1 for any individual i for indoor and outdoor feeds. The total effect of the human population is then calculated through the net probabilities of these four outcomes P_j_, which are calculated as

$$P_{j}=\sum_{i} P_{j,i}P_{f,i}=\sum_{i} P_{j,i}\frac{\alpha_{i}W_{i}}{\sum_{i} \alpha_{i}W_{i}}$$

The P_j_ for indoor feeding sum to 1 and the P_j_ for outdoor feeding sum to 1. The mosquitoes in a cohort are sorted into outcomes A-F, and then the mosquitoes in E and F are sorted according to the P_j_ above. The number of mosquitoes in each successive outcome is calculated from the binomial distribution with the number of unsorted mosquitoes and the conditional probability of the next outcome given that none of the previous outcomes has occurred. The cohort’s numbers are adjusted for dead mosquitoes and eggs are laid depending on the number of survived feedings and the host-associated egg batch sizes. The number of eggs laid for each *Wolbachia* or genetic type becomes a new cohort in the larval queue with zero progress towards emergence.

The number of newly infected mosquitoes is also calculated, and the individual resolution of the human population allows each individual to have an infection-dependent infectivity which is mediated through the effects of any interventions. Let cX_i,transmit_Y_i,transmit_ be the probability that a mosquito surviving a feed upon individual i becomes infected in the absence of interventions, in which X_i,transmit_ is the probability of infection for a fully susceptible mosquito and no transmission-blocking immunity, Y_i,transmit_ is the effect of natural transmission blocking immunity, and c is the effect of mosquito transmission-blocking immunity. This multi-factorial representation allows compatibility of this framework with other modelling approaches. For example, to simulate a Macdonald-style model [1-3] in this framework, X_i,transmit_=1 for infected individuals and 0 for uninfected, Y_i,transmit_=1 so that there is no transmission-blocking immunity, and c is the probability of mosquito infection when feeding on an infectious individual. In compartmental ODE models with transmission-blocking immunity as a human compartment, Y_i,transmit_ is used with X_i,transmit_ and c as before. In a mechanistic intrahost malaria model, the gametocytes and immune factors can be used to directly calculate X_i,transmit_ based on direct-feeding data [4-5]. There is no additional natural transmission-blocking immunity at this time, as immunity reduces the asexual parasitemia driving gametocyte production and incorporates the effects of the inflammatory innate response, both of which affect the value of X_i,transmit_. Y_i,transmit_ and c are then both set to unity in the full malaria model, although c can be reduced for more refractory mosquito species. This framework is created to be flexible enough to be used as the vector component in most malaria models.

The effect of interventions and feeding outcomes upon transmission then depends on indoor versus outdoor feeding and includes the effects of vaccines. Set Z_i,transmit,indoor_ and Z_i,transmit,outdoor_ to be the effect on transmission from humans to mosquitoes of all interventions and feeding outcomes. For outdoor feeding, Z_i,transmit,outdoor_ = (1-E_TBV_) (1- p_humanfeedingmortality_), in which E_TBV_ is the efficacy of all transmission-blocking vaccines present, 0 if not applicable. For indoor feeding, Z_i,transmit,indoor_ = (1-E_TBV_)p_indooroutcome,e_. The probability of mosquito infection will be reduced by returning mortality (1-p_kill,PFV_) if a photonic fence surrounds the village, but apart from that the probability of an outdoor human feeding mosquito surviving and becoming infected is then

$$\sum_{i} P_{f,i}cX_{i,transmit}Y_{i,transmit}Z_{i,transmit,outdoor}$$

The probability of an indoor human feeding mosquito surviving and becoming infected is

$$\sum_{i} P_{f,i}cX_{i,transmit}Y_{i,transmit}Z_{i,transmit,indoor}$$

These are used to calculate the number of infected mosquitoes from the mosquitoes sorted into OUTCOMES E and F for uninfected mosquitoes. The number of newly infected mosquitoes is then removed from the mosquitoes in the cohort calculated to have successfully survived a human feed and create a new cohort of infected mosquitoes with progress=0. If the number of newly infected mosquitoes is larger than the number of successfully human-fed mosquitoes (an unlikely occurrence given the probability of mosquito infection for direct feeding), the number of newly infected mosquitoes is set equal to the number of successfully human-fed mosquitoes.

For the implementation following individual mosquitoes, the probabilities are used to determine a single outcome for each mosquito. When these outcomes are summed over all mosquitoes, the results are approximately equal to the results for the mosquito cohort implementation.

**Calculation of vector infectivity to human population**

The number of infectious mosquitoes that attempt a human indoor or outdoor feed (OUTCOMES E and F) are calculated as outputs N_f,indoor_ and N_f,outdoor_ back to the human population. These are normalized by the effective human population Σ_i_α_i_W_i_, to get the number of indoor and outdoor infectious bites per person in the population. Each sampled individual behaves as a single individual for acquiring infections and the sampling weight W_i_ is only used for an individual’s cross-section to the world, although the relative biting rate α_i_ is still relevant. Thus, if b is the probability of human infection in a fully susceptible individual, Y_i,acquire_ is the effect of natural acquisition-blocking immunity, and Z_i,acquire_ is the effect of interventions on attempted indoor and outdoor bites, then an individual’s rate of new infections R_i_ is calculated as

$$R_{i}=\frac{\alpha_{i}}{\sum_{i} \alpha_{i}W_{i}}bY_{i,acquire}(N_{f,indoor}Z_{i,acquire,indoor}+N_{f,outdoor}Z_{i,acquire,outdoor})$$

Z_i,acquire,indoor_ includes the effects of acquisition-blocking pre-erythrocytic vaccines and interventions which prevent mosquitoes seeking that individual from feeding. Mosquitoes which die during or post-feeding still can cause infection however, so Z_i,acquire,indoor_ = (1-E_PEV_)(p_indooroutcome,c_+ p_indooroutcome,d_ + p_indooroutcome,e_). Z_i,acquire,outdoor_ = (1-E_PEV_), since nets and IRS cannot block an attempted feed or kill a mosquito pre-feeding as for indoor feeds. The probability of an individual acquiring an infection during time step Δt is then 1-e^(-RiΔt)^. If multiples strains are explicitly tracked, infectivity is calculated for each strain.

1. Smith DL, McKenzie FE: **Statics and dynamics of malaria infection in Anopheles mosquitoes.** *Malar J* 2004, **3:**13.

2. Macdonald G: **Theory of the eradication of malaria.** *Bull World Health Organ* 1956, **15:**369-387.

3. Macdonald G: *The epidemiology and control of malaria.* London, New York,: Oxford University Press; 1957.

4. Jeffery GM, Eyles DE: **Infectivity to Mosquitoes of Plasmodium Falciparum as Related to Gametocyte Density and Duration of Infection.** *Am J Trop Med Hyg* 1955, **4:**781-789.

5. Schneider P, Bousema JT, Gouagna LC, Otieno S, van de Vegte-Bolmer M, Omar SA, Sauerwein RW: **Submicroscopic Plasmodium falciparum gametocyte densities frequently result in mosquito infection.** *Am J Trop Med Hyg* 2007, **76:**470-474.
